# Bone Marrow Derived Mesenchymal Stem Cells in Addiction Related Hippocampal Damages

**DOI:** 10.22088/IJMCM.BUMS.7.2.69

**Published:** 2018-06-20

**Authors:** Raheleh Rafaiee, Naghmeh Ahmadiankia

**Affiliations:** 1 *Addiction Research Center, Shahroud University of Medical Sciences, Shahroud, Iran.*; 2 *School of Medicine, Shahroud University of Medical Sciences, Shahroud, Iran.*

**Keywords:** Addiction, hippocampus, neurogenesis, neural stem/progenitor cells, mesenchymal stem cells

## Abstract

The brain is an important organ that controls all sensory and motor actions, memory, and emotions. Each anatomical and physiological modulation in various brain centers, results in psychological, behavioral, and sensory-motor changes. Alcohol and addictive drugs such as opioids and amphetamines have been shown to exert a great impact on brain, specifically on the hippocampus. Emerging evidence has indicated that altered hippocampal neurogenesis is associated with the pathophysiology of neuropsychological disorders including addiction. The addictive drugs impair neurogenesis and undermine the function of neural stem/progenitor cells in hippocampus. This feature was claimed to be one of the underlying mechanisms of behavioral changes in patients with addiction. As the impairment of stem cells’ function has been proven to be the underlying cause of pathologic neuroadaptations in the brain, the administration of stem cell populations has shown promising results for re-modulating of neuronal status in the brain and especially in the hippocampus. Among the different types of stem cells, bone marrow derived mesenchymal stem cells are the most proper candidates for stem cell therapies. In this review article, the recent studies on the effects of addictive drugs on brain neurogenesis, and also the promising potential effects of stem cells in curing addiction related hippocampal damages are discussed.

Addiction is defined as a chronic disease with obligation to take drugs or alcohol, no control on restraining intake, and having negative emotional feeling during withdrawal period. Addiction does not just affect the addict’s life, but also it has a huge burden on the society and economy. It was revealed that the addictive agents have a great anatomical and physiological impact on the brain centers, resulting in psychological, behavioral, and sensory-motor changes(1). It was demonstrated that addictive substances affect dopaminergic pathways which connect the ventral tegmental area to the prefrontal cortex via limbic system in particular in the nucleus accumbens, amygdala, ventral pallidum, and hippocampus (2).

Brain has the ability to produce new neural stem/ progenitor cells (NSPCs) during adulthood. Hippocampus might be the most plastic region of the brain, where granular cells in the dentate gyrus are born in adulthood. The precursors of these cells are placed in the subgranular zone (SGZ), the tissue between hilus and granule cell layer (3, 4). The sensible characteristic of adult-born neurons in the hippocampus is their specific electrophysiological capability for extreme changes required in early stages of maturation. This property is pivotal for formation of memories and further physiological actions (5). The SGZ provides a proper niche for proliferation and differentiation of stem cells in dentate gyrus (6). Astrocytes as important cellular components of SGZ, play an active role in proliferation and neuronal fate commitment of NSPCs (7) through release of molecular signals such as Wnt (8), Ephrin B2 (9), and sonic hedgehog (Shh) (10,11). Moreover, they have been shown to play essential roles in neural cell survival, immune responding, and modulation and metabolism of neurotransmitters (12). Therefore, each stimulant that can affect NSPCs or their niche in the hippocampus could make a vast modification in the memory and behavior. Bulk of studies have found the alterations in adult neurogenesis of hippocampus in neuropsycho-logical disorders such as depression (13), schizophrenia (14), bipolar disease (15), and addiction (16). A large amount of evidence indicates the changes in adult neurogenesis of dentate gyrus in abusing drugs such as opioids (17), amphetamines (18), and alcohol (19).

Addictive drugs and alcohol can regulate NSPCs by a variety of mechanisms. Some of these mechanisms are shared among them (16). For example, they regulate adult neurogenesis by modulating cell cycling pathways (20), and G protein-coupled receptor (GPCR) signaling cascades (21). Moreover, molecules involved in supporting or inhibiting neurogenesis including brain-derived neurotrophic factor (BDNF), interleukin 1 beta (IL1β) or vascular endothelial growth factor (VEGF), could be influenced by some addictive drugs (22). Additionally, they can exacerbate mitochondrial function and invoke oxidative stress (23). There is an evidence that 4-hydroxynonenal (HNE), an aldehydic product of membrane lipid peroxidation, is a key mediator of neuronal apoptosis induced by oxidative stress (24).

Some signaling molecules and pathways such as the mitogen-activated protein kinase (MAPK) signaling pathway, cell cycle regulatory molecules, and microRNAs (miRNAs) which may function independently or act in conjunction with one another have been identified to play important roles in these modulations (25).

Although neurons are the principal targets of drug addiction, it has recently been shown that nearly all drugs of abuse also affect glial cells (26). Astrocytes as the most abundant glial cells in brain (27) are well characterized for their role in the clearance of neurotransmitters, such as glutamate, from the synaptic cleft. Synaptic clearance of glutamate occurs primarily through the glutamate transporter 1 (GLT-1), expressed exclusively on astrocytes (28).

Several lines of evidence indicate that ethanol and other drugs of abuse downregulate the expression of *GLT-1*, leading to excessive accumulation of glutamate in synaptic cleft. Excess glutamate massively stimulates N-methyl-D-aspartate receptors (NMDARs). Massive stimulation of NMDARs leads to an excessive cellular influx of ions, particularly calcium, causing the activation of proteases, phospholipases, and endonucleases that end in cellular death (29). This form of neuronal death caused by hyperactivity of excitatory amino acids, mainly glutamate, is named excitotoxicity. It is one of the other mechanisms that have been proposed for alcohol and drug induced brain damages (30). Drug relapse observed for alcohol and other drugs is causally associated with the existence of high levels of extracellular glutamate (31).

Addictive drugs also activate microglia and

astrocytes via toll-like receptor 4 (TLR4), leading to the release of pro-inflammatory cytokines, and reactive oxygen species which in turn, promote neuronal death in hippocampus and other brain regions (32).

In the first part of this review, the effects of drug and alcohol abuse on neurogenesis are discussed, then we provide an overview of the promising effects of bone marrow derived mesenchymal stem cells (BM-MSCs) for the treatment of addiction related hippocampal damages.

## Drug associated alterations in neurogenesis in hippocampus

The principal centers that are directing the feelings and are affected by addictive drugs are hippocampus and medial prefrontal cortex. It has been indicated that the behaviors of drug seeking and relapsing to drug abuse are mediated by these centers (33, 34).

Animal studies showed that self-administration of drugs attenuates neurogenesis in dentate gyrus (1, 35-43). The examples of these results include the decrease in proliferation and differentiation of NSPCs in dentate gyrus following nicotine self-administration (1), and attenuation in proliferation of NSPCs after heroin (38) and cocaine self-administration (41, 43). Access to cocaine augments the differentiation of dentate NSPCs, but does not affect their survival (39). Acute cocaine exposure was shown to cause a significant increase in oxidative stress in human NSPCs, which was followed by drastic apoptotic effects (44). Further studies demonstrated that although the proliferating cells in both SGZ and SVZ of rats decreased after 3 weeks of cocaine self-administration, the effects were reversed by 4 weeks of withdrawal (39).

Methamphetamines as another drug have non-linear effect on the dentate gyrus stem cells. In the case of daily self-administration of methamphetamine, the survival, proliferation, and differentiation of progenitors are decreasing. However, intermittent access raises proliferation and differentiation, but this type of increase in the population of immature neuronal cells does not alter survival and neurogenesis of hippocampal progenitors, perhaps because of opposing effects on proliferation of late progenitors and differentiation of post-mitotic neurons (36). Methamphetamine delays cell cycle progression from G0/G1-to-S phase. This effect could be due to the down-regulation of cyclin E, and to the decrease of epidermal growth factor receptor (EGFR) and ERK1/2 phosphorylation which are involved in cell proliferation progression (45). Several studies using animal models have shown the involvement of oxidative stress and excitotoxicity in the neurotoxicity produced by methamphetamines (46). Neurogenesis in the dentate gyrus decreased markedly in amphetamine-treated rats following four weeks of withdrawal from amphetamine (47).

Neuroinflammation is also associated with the chronic use of addictive drugs including cocaine, opiates, marijuana, and methamphetamine (48).

It is vital to indicate that all above studies show a correlation between daily drug intake and alteration in neurogenesis. As the amount of drug intake increases, the pathologic effects are more on the dentate gyrus neurogenesis.

## Alcohol-associated alterations in the neurog-enesis in hippocampus

Alcohol abuse often leads to the alcohol use disorder (AUD) that has great deteriorating impacts on the brain. Excessive use of alcohol results in progressive neurodegeneration in brain that also accelerates AUD (49). Alcohol abuse causes general changes in white and gray matters in the central nervous system (50-52); nevertheless, some brain centers are more affected by alcohol abuse. Ethanol neurotoxicity greatly disturbs hippocampus and frontal cortex (52, 53). The altered integrity of hippocampus in alcoholics leads to abnormal cognitive functions and psychopathological actions (54, 55), which further results in AUD development (55, 56). Studies on human and animal models generated valuable information about the effects of alcohol abuse on the brain. Examples of alcohol effects on the brain structure and function include cell loss in corticolimbic regions (57, 58), decrease of the complexity of dendritic branching (59), and alterations in dendritic spine structure (60, 61). Moreover, alcohol self-administration in animal models resulted in decrease in survival, proliferation and differentiation of NSPCs in dentate gyrus in the hippocampus (37, 62, 63). Hippocampus is the most pathologically affected area of brain by chronic ethanol intake. Ethanol can significantly alter the expression of genes involved in neural differentiation pathways including axon guidance, hedgehog signaling, TGF-b signaling,; cell adhesion molecules, and Wnt signaling in differentiating human neuronal stem cells (64). Cumulative evidence indicates that ethanol activates microglia and astrocytes via TLR4, which can be evidenced by specific morphological changes including increased length and thickness of primary processes (65). This activation promotes the release of pro-inflammatory cytokines that in turn, promote neuronal death in the hippocampus and other brain regions (32).

Withdrawal from ethanol exposure enhanced cell proliferation in the hippocampus, resulting in initial microglial proliferation followed by the production of immature neurons and eventual neurogenesis (66, 67). The mechanisms underlying ethanol withdrawal-induced aberrant neurogenesis in the dentate gyrus are not yet completely elucidated.

## The role of stem cells in treatment of addiction related hippocampal damages

What we summarized in above topics are brief description of the effects of drugs and alcohol in addicted patients on adult neurogenesis. The NSPCs are introduced as the main affected cell population in addiction. In this part, the potential of BM-MSCs for treating injuries to the brain with focus on addiction-derived alterations is discussed.

Stem cells are primary cells with self-renewal and differentiation ability (68). The most famous multipotent stem cells are MSCs which are recognized by their ability to differentiate into adipocytes, chondrocytes and osteocytes, and their plastic adherence (69). Available results from clinical studies support the overall safety of cell therapy using MSCs (70). The other extreme valuable characteristic of MSCs is the immunomodulatory effect of these cells. This feature is so beneficial in disorders with inflammatory components (71). The MSCs are also non-immunogenic; therefore, they can be easily obtained from allogeneic sources as they are not provoking lymphoproliferative responses (72, 73).

MSCs have been applied in neurological degenerative disorders such as Parkinson’s disease (74), Alzheimers disease (75), amyotrophic lateral sclerosis (76), and traumatic and ischemic brain injuries (77, 78). BM-MSCs have been differentiated into neuron-like and glial cells both *in vivo* and *in vitro *(80-82). There is an evidence for crossing the blood barrier of adult rat brain by human adipose-derived mesenchymal stromal cells (82). The BM-MSCs have also the potential to pass through blood brain barrier in hypoxic-ischemic encephalopathy animal model (83).

A considerable body of evidence has revealed the potential of BM-MSCs secretome to modulate neuronal survival and differentiation. This effect is attributed to BM-MSCs secretion of BDNF, GDNF (glial derived neurotrophic factor), and basic fibroblast growth factor (bFGF). These neuro-regenerative effects were accompanied by the improvement in animals’ memory and motor behavior (84). It was revealed that MSCs increase hippocampal neurogenesis and neuronal differentiation by enhancing the Wnt signaling pathway (85). Stromal cell-derived factor 1 (SDF)-1α as another cytokine released from BM-MSCs is associated with neural protection through anti-apoptotic based mechanisms (86). Additionally, the secretion of BDNF *in vivo* by BM-MSCs, was correlated with the activation of endogenous stem cells (87). Secretion of these factors by MSCs not only protects neurons from further degeneration and enhances neurogenesis, but also acts as immune response modulator. The overall expression of pro-inflammatory cytokines, such as IL-1β, IL-2, IL-12, tumor necrosis factor alpha (TNF-α), and interferon γ (INF γ) decreased after MSC transplantation. It has been shown that MSCs and their released cytokines and growth factors protect hippocampal neurons from oxidative stress and synapse damage induced by amyloid-β oligomers (88). Conditioned medium from MSCs also protect CNS neurons against glutamate excitotoxicity by inhibiting glutamate receptor expression and function (89).

Besides soluble growth factors and cytokines, MSCs also secrete microvesicles and exosomes containing mRNAs and/or miRNAs, which are believed to mediate cell-to-cell communication (90). Exosomes secreted by BMSCs in vitro not only mediate communication with neurons and astrocytes, but also regulate neurite outgrowth by transfering miRNA (miR-133b) into neural cells (91).

**Fig. 1:**
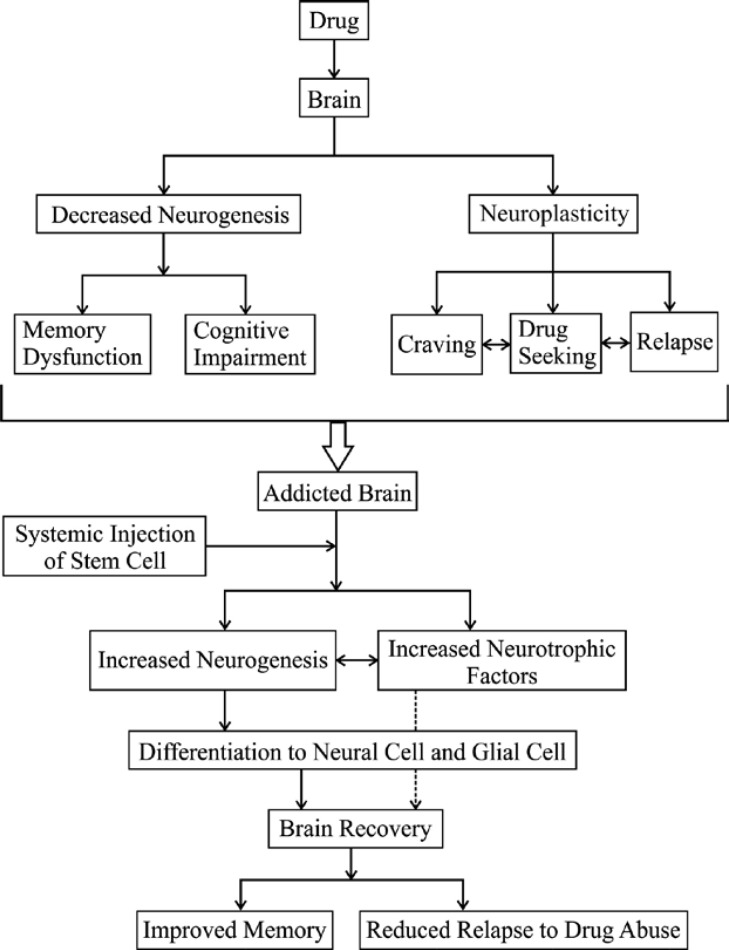
Schematic representation of drug effects on brain, and the benefits of stem cell injection

MSCs have shown therapeutic effects on the brain pathologies. A crucial finding in mental illnesses like bipolar disorder, major depression, and schizophrenia is the disturbance of neurotrophic factors and immunomodulatory systems in the brain. The pro- inflammatory cytokines such as TNF-α, IL-6, and IL- 2 are increasing and BDNF is decreasing in such disorders (92-94). Furthermore, as we specified above, there are alterations in anatomy and neurogenesis of hippocampus in psychological disorders and in individuals with addiction to drugs and alcohol (95-97). There are few studies with the aim of evaluating the effects of MSCs in psychiatric models. Application of MSCs in an animal model of depression has led to the improvement in hippocampal neurogenesis and depressive behaviors (98). In addition, intra-hippocampal injection of MSCs improved neurogenesis with no behavioral changes in rats, which indicates the safety of MSCs transplantation in brain (99). The secretion of neurotrophic factors from MSCs and the immunomodulatory function of these cells could be the possible regenerative mechanism of these cells on neurogenesis (100, 101).

A brief description of the effects of drug on brain and the benefits of stem cell injection on addicted brains is represented in figure 1.

Yang et al. transplanted labeled BM-MSCs into the hippocampus of alcohol-associated dementia animal model. Evaluation of the behavior and hippocampus structure of the injected rats revealed that their learning and memory function were enhanced, alcohol-induced hippocampal injures were inhibited in histological examinations, the number of apoptotic neural cells was decreased, and the activity of total superoxide dismutase was increased in the hippocampus. Transplantation of BM-MSCs also increased the level of BDNF protein (102).

In a study by Israel et al, alcohol drinking rats were injected with human BM-MSCs and adipose tissue-derived MSCs intra- cerebro- ventricularly (ICV). The results showed that injected MSCs survived and became attached to cerebral ventricles. Transplanted MSCs reduced 24-h alcohol intake and also blocked alcohol relapse-like drinking induced in the alcohol deprivation effect condition (103). Ezquer et al. showed that administration of a single dose of human BM-MSC-spheroids, whether ICV or intravenously, greatly reduced neuro-inflammation, and inhibited chronic alcohol intake and relapse-like drinking. Administration of BM-MSC-spheroids also markedly increased the levels of the GLT-1, leading to inhibition of relapse. It was also revealed that human BM-MSC-spheroid administration in alcoholic rats fully normalized astrocyte activation, and decreased *MCP1 *expression in the hippocampus, suggesting a potent anti-inflammatory effect of BM-MSC-spheroids. Furthermore, oxidative stress was normalized by MSC- spheroid administration (104).

Therapeutic promises of BM‐MSCs have been overshadowed by concerns regarding their limited homing potential or migration to non-target sites (105, 106). Although, extensive investigations have provided significant potential for enhancing targeted stem/progenitor cell homing (107-110) there are some limitations that make it difficult to apply these findings in clinics, especially in neurodegenerative disorders.

## Conclusion

Several studies indicated the damaging effects of drug and/or alcohol abuse on the brain neuroanatomy and function. Experiments have revealed that addiction leads to impairment in adult neurogenesis in behavioral centers of brain including hippocampus and medial prefrontal cortex. Numerous experiments on animal models have shown the effects of addictive drugs such as morphine, cocaine, methamphetamines, and alcohol on the proliferation, survival and differentiation of progenitor cells in the hippocampus. Animal studies showed promising results after hippocampal transplantation of BM-MSCs in psychological disorders e.g. depression and alcohol abuse. More studies on the stem cell therapy of psychological defects related to addiction are required.

## Conflict of interest

The authors declare no conflict of interest.
